# Synergistic Impact of Copper Nanoparticles Functionalized with Magnetic Chitosan on the Enhanced Adsorptive Sequestration of Metformin Diabetic Drug from Environmental Samples

**DOI:** 10.3390/polym17223046

**Published:** 2025-11-17

**Authors:** Khairia M. Al-Ahmary, Abeer H. Aljadaani, Amr A. Yakout

**Affiliations:** 1 Department of Chemistry, College of Science, University of Jeddah, Jeddah 21589, Saudi Arabia; 2 Chemistry Department, Faculty of Science, Alexandria University, Alexandria 21321, Egypt

**Keywords:** copper nanoparticles, magnetic chitosan, metformin, pharmaceuticals, reusability

## Abstract

Pharmaceutical residues, including a wide range of therapeutic drugs, have been increasingly reported in drinking water sources worldwide, raising environmental concerns due to their potential impact on aquatic ecosystems. Among the available treatment approaches, adsorption has emerged as one of the most reliable methods for eliminating these pollutants. In the present study, metformin was effectively removed from water using a nanocomposite adsorbent consisting of copper nanoparticles anchored onto magnetic chitosan (Cu@MCS). The removal of metformin by Cu@MCS was governed by several mechanisms: surface complexation with copper species, electrostatic interactions, hydrophobic associations between the drug’s methyl groups and magnetite, and hydrogen bonding between metformin’s amino groups and oxygenated functional groups of chitosan. The structural and surface properties of the nanocomposite were characterized through FTIR, XPS, XRD, SEM, and HRTEM analyses. Key experimental factors, such as initial drug concentration, contact time, pH, and ionic strength, were systematically optimized to maximize adsorption efficiency. Adsorption data closely followed the Langmuir isotherm model, with a maximum capacity (*q*_m_;) of 52.91 mg·g^−1^ at 298 K. Regeneration tests demonstrated excellent reusability, showing only a 3.7% decline in performance after six adsorption–desorption cycles. The Cu@MCS material also proved effective in removing metformin from diverse real water samples, including river water, wastewater, bottled water, and tap water. A notable advantage of this nanosorbent is its magnetic separability, which enables straightforward recovery from solution, even at low contaminant levels and with large sample volumes. These results underline the potential of magnetic chitosan-based nanocomposites as fast, efficient, and reusable adsorbents for the removal of pharmaceutical contaminants from aquatic systems.

## 1. Introduction

Pharmaceutical residues have increasingly been detected in drinking water sources worldwide, raising concerns about their potential risks to aquatic ecosystems and human health [1,2,3,4]. Large amounts of therapeutic drugs enter wastewater and groundwater treatment facilities through human excretion, sewer leakage, and the disposal of expired medications [5,6,7]. These pollutants are persistent, bioactive, and often remain unmetabolized, making their presence in water bodies an emerging environmental issue of global importance.

Among these contaminants, metformin (1,1-dimethylbiguanide), a first-line treatment for type 2 diabetes and polycystic ovarian syndrome, is one of the most widely consumed pharmaceuticals, with more than 150 million users annually [8,9,10]. Approximately 70% of ingested metformin is excreted unchanged in urine, leading to frequent detection in sewage effluents and surface waters at concentrations ranging from micrograms to milligrams per liter in different countries [11,12,13,14,15,16,17]. Its continuous release into aquatic systems raises ecological and health concerns, including endocrine disruption in fish and possible effects on human vitamin B12 metabolism [18,19]. Yet, no regulatory limits for metformin in drinking water have been established, underscoring the urgency of developing effective remediation strategies.

Various treatment methods, such as coagulation [20], biological degradation [21], photocatalysis [22], catalytic ozonation [23], electrochemical oxidation [24], and adsorption [25,26,27], have been investigated for pharmaceutical removal. Among these, adsorption stands out due to its simplicity, efficiency, and wide availability of sorbents [28]. Several adsorbents, including magnetic chitosan [29], activated carbon [30,31], magnetic carbon composites [32,33], silica–alumina [34], biochar [35,36], and natural clay [37], have been applied for metformin removal. Chitosan is attractive because of its abundance, eco-friendliness, and multiple amino and hydroxyl groups that enable strong binding with pollutants [38,39,40,41]. However, conventional chitosan-based sorbents often suffer from limited separation efficiency, poor stability, or aggregation issues, which restrict their large-scale applicability [42,43,44,45].

Although many adsorbents have been explored, most are unsuitable for real industrial applications due to limited separation efficiency. Magnetic separation offers distinct advantages, including low cost and high efficiency, by employing carriers with a magnetic core for strong response and a polymeric shell for surface functionality [46,47,48]. Nanosized carriers further outperform microsized supports because of their higher surface area and reduced diffusion resistance [49,50]. Magnetite (Fe_3_O_4_), with its excellent magnetic properties, chemical stability, and biocompatibility, has thus been widely used to impart magnetic functionality. Chitosan, with abundant amine and hydroxyl groups, provides strong interaction sites, and when magnetically modified, can be easily separated from aqueous systems under an external field [51]. Such magnetically functionalized chitosan systems have shown promise in removing heavy metals [52,53,54,55,56], dyes [57,58], and aflatoxins [59], as well as serving in green catalysis [60,61] and drug delivery applications [62,63]. Building on this foundation, our ongoing research focuses on further enhancing chitosan- and graphene-based nanocomposites for efficient removal of emerging pollutants from water.

**Figure 1:**
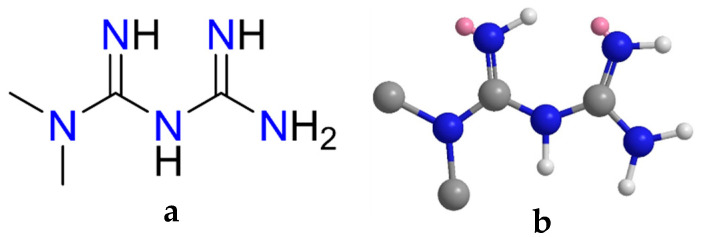
The (**a**) 2D and (**b**) 3D structures of metformin.

In this study, we address these limitations by developing a copper-functionalized magnetic chitosan nanocomposite (Cu@MCS) as a novel nanosorbent for the rapid and efficient adsorption of metformin (Figure 1) from water. The synergistic role of Cu nanoparticles is to prevent aggregation of magnetic chitosan, enhance surface area, and promote strong Cu–metformin surface interactions, thereby improving adsorption performance. To the best of our knowledge, this is the first report demonstrating the application of Cu@MCS for metformin and pharmaceutical wastewater treatment, combining high efficiency, rapid kinetics, and excellent reusability.

## 2. Experimental Section

### 2.1. Materials and Reagents

Sodium chloride (NaCl), L-ascorbic acid, copper nitrate trihydrate (Cu(NO_3_)_2_·3H_2_O), FeSO_4_, FeCl_3_, and methanol were analytical reagent grade (Sigma-Aldrich, St. Louis, MO, USA). We purchased 99.99% pure metformin HCl from (Selleck Biotechnology GmbH, Cologne, Germany). Sigma-Aldrich provided chitosan powder. By dissolving the required amount of metformin, stock standard solutions were generated (500 mg/L), which were then stored at 4 °C. By diluting the stock solutions with Milli-Q water, the proper concentrations of working solutions were made each day.

### 2.2. Instrumentation

A Nicolet 400 Fourier transform infrared spectrometer was used to perform the FTIR measurements. Using a quartz cuvette with a path length of 1 cm, the Labomed Inc. UVD-3500 UV-Vis double-beam spectrophotometer (Culver City, CA, USA) was used to estimate the metformin concentration. pH values were adjusted using a combined glass electrode and a calibrated pH meter. X-ray powder diffractometer with a Cu-Kα radiation source (k = 1.5418 Å) (D/MAX-2550-Rigaku, Tokyo, Japan). High-resolution transmission electron microscopy (HRTEM) with JEOL JEM-2100V (JEOL, Peabody, MA, USA) and scanning electron microscopy (SEM) with JEOL JSM-6010LV (JEOL, Peabody, MA, USA) were used to examine the morphology of MCS and Cu@MCS nanocomposites.

### 2.3. Preparation of Cu@MCS Nanoparticles

A nanoparticle-sized Cu-functionalized magnetic chitosan nanocomposite was created by mixing Cu-NPs with magnetic chitosan (MCS) in a ball mill. To make the MCS nanoparticles, ferrous and ferric ions were chemically co-precipitated with NaOH and chitosan in a solution that was then hydrothermally treated [64,65]. In a nutshell, 2.0 g of chitosan was dissolved in 100 mL of a 2.0% acetic acid solution. In a 1:2 molar ratio, FeSO_4_ and FeCl_3_ were dissolved. Then, 15 mL of 30% NaOH solution was added dropwise while stirring constantly under argon to chemically precipitate the resultant solution at 40 °C. Separation was achieved by centrifugation many times in water and ethanol after heating the suspension to 90 °C and maintaining continuous stirring for 1 h. Then, the particles were dried in a vacuum at a temperature of 70 °C. A basic precursor for Cu was used in a 0.1 *M*-Cu(NO_3_)_2_ solution, L-ascorbic acid served as an antioxidant, and PVP was used as a stabilizing and reducing agent. With the help of a catalyst, NaOH (0.2 *M*), the pH was brought down to 12. A 100 mL stainless-steel autoclave lined with a Teflon container was used to heat the combination to 190 °C for five hours after it had been carefully placed inside. Following centrifugation, the brownish Cu nanoparticles were washed with a mixture of ethanol and water and then dried in an oven. As a last step, the black Cu@MCS nanocomposite was created by carefully blending the Cu nanoparticles with the black MCS nanosheets using a stainless-steel ball mill set to 25 Hz for 30 min.

### 2.4. Batch Experiments for the Adsorption Removal of Metformin by Cu@MCS Nanocomposite

In a centrifuge tube, 10 mg of Cu@MCS nanoparticles were exposed to a particular concentration range (10–400 mg∙L^−1^) of metformin solution. The suspending solutions had been enclosed in aluminum foil and ultrasonicated for two minutes, and the pH was brought down to 6.0 by 0.05 *M* HCl or NaOH to prevent any photodegradation of metformin. The solution was stirred at 250 rpm using a thermostatic shaker. Following separation, a typical calibration curve of various metformin concentrations was used to quantify the clear solution of metformin spectrophotometrically at a wavelength of 235 nm [66]. To examine the effects of pH, the suspension of the nanocomposite and metformin was changed from 2 to 11. Then, 10 mg of the Cu@MCS nanoparticles were introduced to 20.0 mL of metformin solutions at varying starting concentrations (10.0, 20.0, and 40.0 mg L^−1^) to examine the adsorptive removal efficiency kinetics. A series of measurements was made of the metformin concentration at various intervals of time (1–40 min). By dissolving varying concentrations of NaCl, the effect of ionic strength (0–50 mM) on the effectiveness of metformin removal was investigated. Equations (1) and (2) [67,68] were used to determine the metformin removal efficiency (%*R*) and capacity of adsorption (*q_e_*) in mg∙g^−1^.

(1) $$\%R=\frac{C_{o\;}-C_{e\;}\;}{C_{o\;}}\times 100$$

(2) $$q_{e}=\frac{C_{o\;}-C_{e\;}\;}{m}\times V$$

*C_o_* and *C_e_* denote the metformin original and final concentrations (mg∙L^−1^), respectively, *m* is the mass of the adsorbent (g), and *V* represents the solution volume (L).

## 3. Results and Discussion

### 3.1. Surface Morphology and Characterization

**Figure 2:**
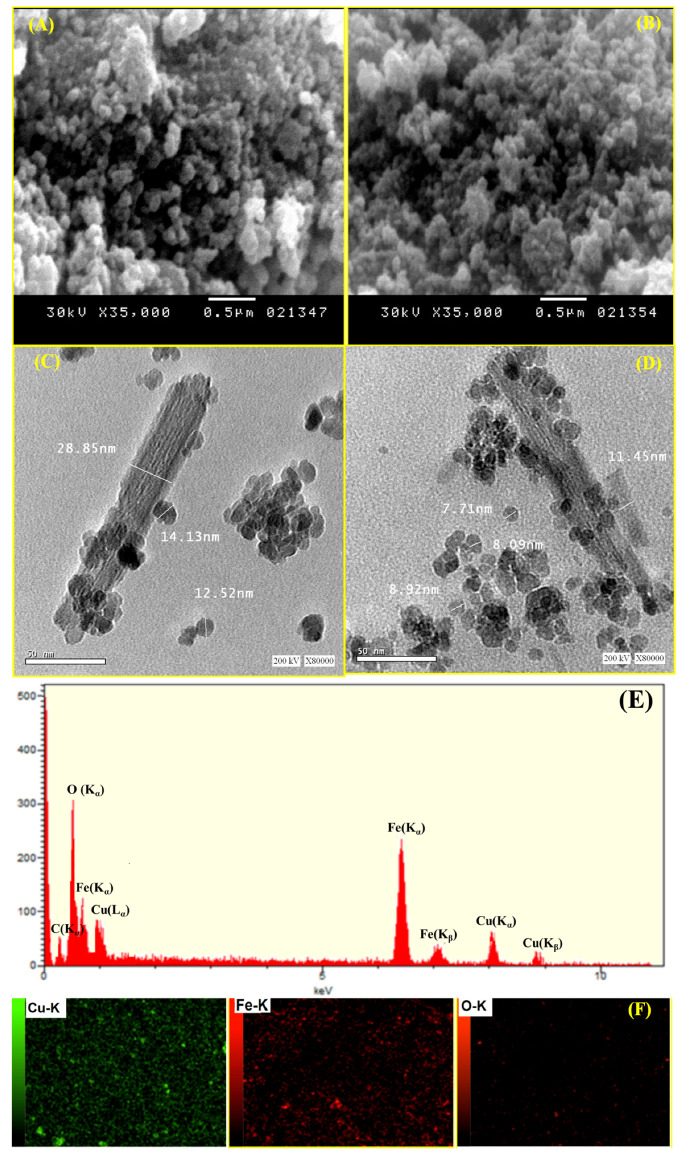
SEM (**A**,**B**) and HRTEM (**C**,**D**) morphologies of MCS and Cu@MCS nanocomposite; EDS (**E**) and mapping EDS (**F**) of Cu@MCS nanocomposite.

**Figure 3:**
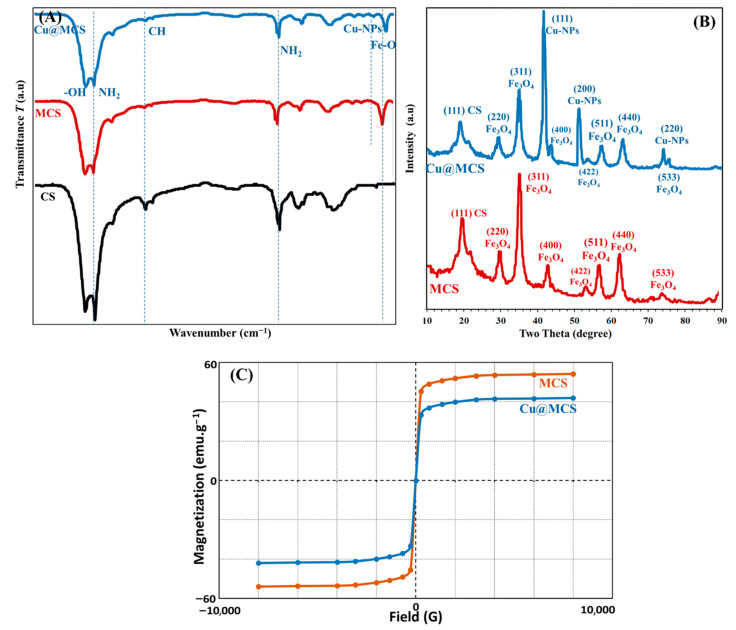
FTIR spectrum (**A**), XRD (**B**), and VSM (**C**) of Cu@MCS nanocomposite.

Using HRTEM and SEM, the surface morphologies of MCS nanosheets and the Cu@MCS nanocomposite were investigated; the resulting images are shown in Figure 2A–D. A vertical, compacted stack morphology and wrinkled structure appeared in the scanning electron micrograph of MCS (Figure 2A). Agglomeration of MCS with wrinkled films was reduced, to some degree, by the immobilized copper nanoparticles (Figure 2B). Figure 2C,D show HRTEM images of MCS and Cu@MCS, which reveal dark patches on the MCS sheets, which are magnetite and Cu-NPs moieties. The 3D Cu@MCS nanocomposite’s thicker, rougher, and more inconsistent surface, as well as its more random 3D porous arrangement, demonstrates that the MCS surface was effectively doped with Cu-NPs. The Cu@MCS nanocomposite has an average diameter of 25.3 ± 1.5 nm. The N_2_ adsorption/desorption hysteresis loop of the Cu@MCS nanocomposite is shown in Figure S1. Hysteresis loop analysis shows that the Cu@MCS nanocomposite has a microporous structure, which causes a considerable increase in pressure at relatively low pressures (P/Po < 0.05 atm). In addition, the presence of mesopores is shown by the BET isotherm, which shows type IV with an H4 hysteresis loop. The specific surface area and total pore diameter were measured to be 414.33 m^2^∙g^−1^ and 5.40 nm, respectively. Figure 2E,F show the results of the elemental analysis, which verify the chemical purity of the porous Cu@MCS nanocomposite, as well as the presence of magnetite and Cu dopant. The EDX spectrograph of the nanocomposite shows three different peaks of Cu nanoparticles (Figure 2E). The first peak is at 0.93–1.21 keV (L_α1_), the second and largest peak is at 7.95–8.31 keV (K_α_), and the third peak is at 8.78–8.93 keV (K_β_). Additionally, at 0.58–0.67, 6.28–6.71, and 6.92–7.22 eV, the three distinctive peaks of the magnetite nanoparticles are detected. Figure 2F shows the results of the EDS mapping, which indicate the distribution of Cu-NPs and Fe, O magnetite throughout the Cu@MCS nanoparticles. The EDS spectra confirm the presence of Cu loading on the magnetic chitosan nanoparticles. Figure 3A shows the Fourier transform infrared spectra of the MCS and Cu@MCS nanostructures. At the 3471–3512 cm^−1^ range, the stretching vibration of O-H in MCS and Cu@MCS was identified. The C-H stretching vibrations of the -CH_2_- groups in the chitosan polymer backbone are connected to the absorption bands at 2927–2944 cm^−1^ in MCS and Cu@MCS. At 3414–3417 and 1615–1627 cm^−1^, the typical biosorption peak of primary amine (-NH_2_) is visible. C-O bond stretching vibration is shown by the bands at 1030–1075 cm^−1^. At 607.5 cm^−1^, the immobilized Cu-NPs onto the Cu@MCS nanocomposite show their most unique peak, whereas the peak at 581.4 cm^−1^ is assigned to the stretching vibration of the Fe-O in the magnetite. Cu nanoparticles are shown to be immobilized on MCS nanostructures by FTIR spectroscopy. In Figure 3B, XRD results validate the Cu@MCS nanocomposite’s structure. The three diffraction peaks observed in Cu@MCS, namely at 43.94° (111), 51.21° (200), and 75.02° (220), are quite close to the typical Cu card (JCPDS Card: 04-0836) [66]. The CS sheets are responsible for the extra broad (111) peak at around 19.91–20.02° [67]. The magnetite nanoparticles in MCS and Cu@MCS exhibit six distinct diffraction peaks, which align with the standard Fe card (JCPDS Card: 19-0629) at 30.20–30.37° (220), 35.53–35.60, 43.94–43.40, 53.20–53.63° (511), 62.74–62.77°, and 73.38–74.31° (533) [68]. The nanocomposite’s XRD spectrogram displays no extra peaks, suggesting that the fabricated nanocomposite is highly pure.

**Figure 4:**
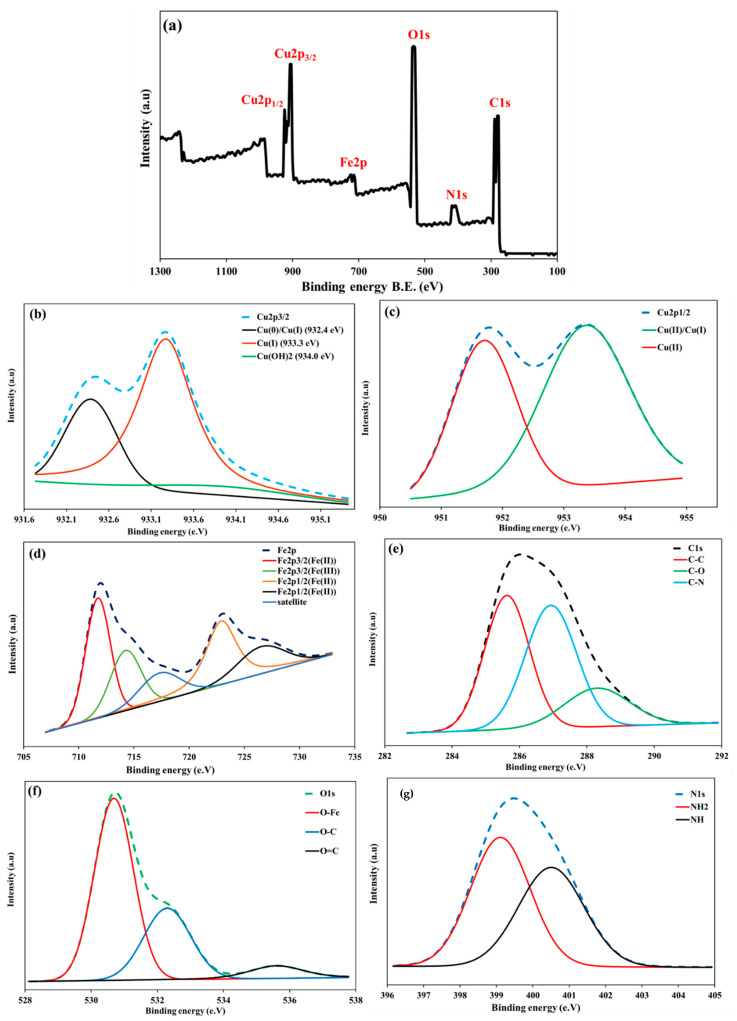
XPS spectrum of full spectrum for Cu@MCS nanocomposite (**a**), high-resolution scan of Cu2p^3/2^ (**b**), Cu2p^1/2^ (**c**), Fe2p (**d**), C1s (**e**), O1s (**f**), and N(1s) (**g**).

Figure 3C depicts the magnetic hysteresis loop of Cu@MCS, which demonstrates its superparamagnetic behavior at ambient temperature. In other words, it is the ability to react to a magnetic field without keeping any magnetism once the field is removed. The VSM was used to characterize the magnetic behavior of magnetic nanoparticles. There is a saturation magnetization (Ms) of 42.11 emu/g in Cu@MCS. Accordingly, the nanocomposite can be isolated from the solution by using an external field. XPS measurements were also used to identify the elemental chemical states, such as the oxidation states and elemental composition, in the Cu@MCS nanocomposite that correspond to the characteristic peaks of Cu2p, Fe2p, O1s, and C1s (Figure 4). With a 20-eV spin-energy gap, the two prominent XPS peaks in the spectrum for Cu2p seen in Figure 4a, which correspond to Cu2p_1/2_ and Cu2p_3/2_, respectively, and have binding energies of 953.2 and 933.1 eV, confirm the doped Cu-NPs [69]. Cu2p_3/2_’s deconvoluted XPS spectra (Figure 4b) display three appropriate wide bands at binding energies of 932.2, 933.3, and 934.0 eV, which are associated with Cu(0)/Cu(I), Cu(II), and Cu(OH)_2_, respectively [69]. The deconvoluted XPS spectra of Cu2p^1/2^ (Figure 4c) reveal two fitted wide bands at binding energies of 951.7 eV and 953.4 eV, corresponding to Cu(II)/Cu(I) and Cu(II), respectively [69]. The analyzed peaks of Cu2p_1/2_ and Cu2p_3/2_ in the XPS spectrum validate the effective functionalization of Cu-NPs. The Fe2p XPS spectra of the nanocomposite (Figure 4d) show binding energies of 722.6 and 726.6 eV for the Fe2p_1/2_ for the Fe(II) and Fe(III), respectively, as well as 711.8 and 714.3 eV for the Fe2p_3/2_ for the Fe(II) and Fe(III) [70]. Three fitting peaks at binding energies of 285.6, 286.9, and 288.4 eV are visible in the deconvoluted XPS spectra of C1s (Figure 4e), and they may be associated with C–C, C–N, and C–O chemical bonds [71]. Three oxygen contributions can be seen in the high-resolution spectrum of O1s. The first peak, located at a binding energy of 530.6 eV, is attributed to the oxygen atoms in the Fe_3_O_4_ lattice. The other two peaks, located at binding energies of 532.3 and 535.6 eV, can be attributed to the O–C and O=C groups, respectively (Figure 4f). Moreover, the presence of chitosan nitrogen on the nanocomposite surface would be shown by the N1s XPS spectrum. The N1s’ two peaks, which represent the NH_2_ and N-H of the amine group, are situated at 399.1 eV and 400.5 eV, respectively (Figure 4g).

### 3.2. Impact of pH

**Figure 5:**
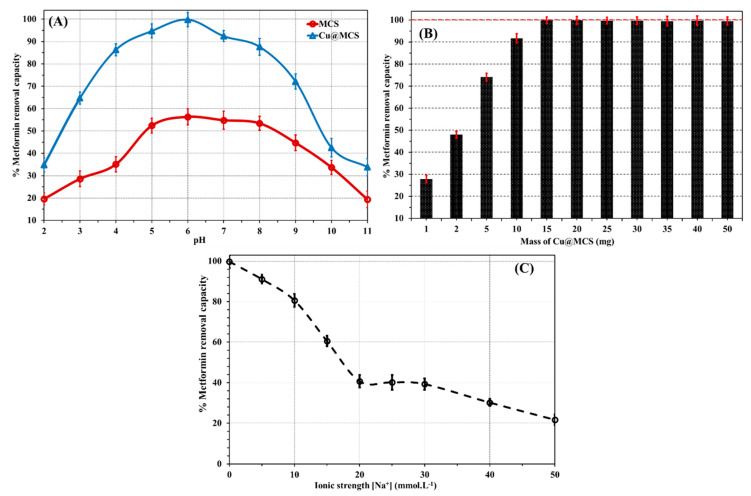
(**A**) Impact of initial pH, (**B**) mass dose of Cu@MCS nanocomposite, and (**C**) solution ionic strength on the removal of metformin at the optimum conditions (initial metformin concentration is 20 mg L^−1^; *T* = 298.15 *K*; nanocomposite dosage is 15 mg, mean ± standard deviation, *n* = 3).

Because of the interaction between the functional groups in metformin and those on the surface of the Cu-immobilized magnetic chitosan nanocomposite, the pH level influences how well metformin is adsorbed by the Cu@MCS nanocomposite. Figure 5A presents the findings of this investigation. At pH 6.0, the sorption capacities attain their maximum values after increasing significantly during the studied pH range of 2–11.

After that, they sharply decline at alkaline pHs. Metformin has two methyl substituents at position 1 and belongs to the biguanide class of guanidines. The functional groups of metformin include primary, secondary, and tertiary amines, as well as two imines (C=N−H). There are two acid dissociation constants for metformin, which are 2.8 and 11.5 [72]. This means that it mostly exists at physiological pH levels as a hydrophilic cationic species or predominantly occurs in the ionized state at pH levels ranging from 5 to 9 [72]. According to its molecular structure and p*K*_a_ values, metformin exhibits a positive charge at low pH, a zwitterionic charge at neutral to moderately acidic pH, and a negative charge at basic pH. At acidic levels of pH (pH < 4.0), the ability to adsorb is thought to be due to H-bonding, electrostatic, and hydrophobic interactions. In hydrophobic interaction, the two methyl groups (on the 3°-N-atom) of metformin interact with the magnetite in MCS. In the electrostatic interactions, the cationic/protonated nitrogen functional groups (1°, 2°, and 3° amines and two imine groups) in metformin interact with the negatively charged oxygen atoms in magnetite and the hydroxyl groups of chitosan in MCS. Aside from the H-bonding interactions between the many hydroxyl and amine groups in MCS and the N-containing functional groups in metformin, the main reason for the increase in adsorption capacities at neutral to moderate pH levels (4.0–10) is the surface complexation interaction between the Cu-NPs and the two imines, as well as the 1° and 3° amine groups in metformin. On the other side, at basic pH values (pH > 10), the reduction in the H-bonding, electrostatic interactions, and the absence of surface complexation between Cu-NPs and N-functional groups in metformin are the main factors responsible for the experimental drop in the identified values of adsorption capacity. The weakness of these interactions can be attributed to the charged ammonium groups in metformin deprotonating at basic pH values.

### 3.3. Impact of Mass and Ionic Strength

The influence of ionic strength on the adsorptive removal efficiency was assessed by adding varying contents of NaCl to the suspended solution of metformin and Cu@MCS nanocomposite. The findings of this investigation are displayed in Figure 5B. The same pattern shows a progressive decline in the removal efficiencies of metformin by Cu@MCS for a range of NaCl concentrations of 0–50 mM at 20.0 mg L^−1^ metformin. At 50 mmol L^−1^ NaCl, the metformin uptake efficiency values dropped by 78.1%. Electrostatic screening phenomena were used to explain this behavior. The solution’s increased ionic strength would prevent the electrostatic interactions necessary for metformin’s surface complexation with copper nanoparticles. The effect of the nanocomposite mass dose (1–50 mg) for metformin removal with Cu@MCS was performed at pH 6 for a 20 min contact time and an initial concentration of 20 mg∙L^−1^ (Figure 5C). The results showed that increasing the dose from 2 mg to 15 mg increased the percentage of metformin uptake on Cu@MCS from 48 to 92%. Raising the Cu@MCS dose would make abundant active sites of Cu@MCS available to attract more metformin molecules from the solution [73]. The recorded metformin removal efficiencies remained constant at mass dosages higher than 15.0 mg. Therefore, we selected a mass dosage of 15.0 mg of nanocomposite for the subsequent studies.

### 3.4. Cu-Supported Nanoparticles’ Impact on Metformin Uptake by the Cu@MCS

**Figure 6:**
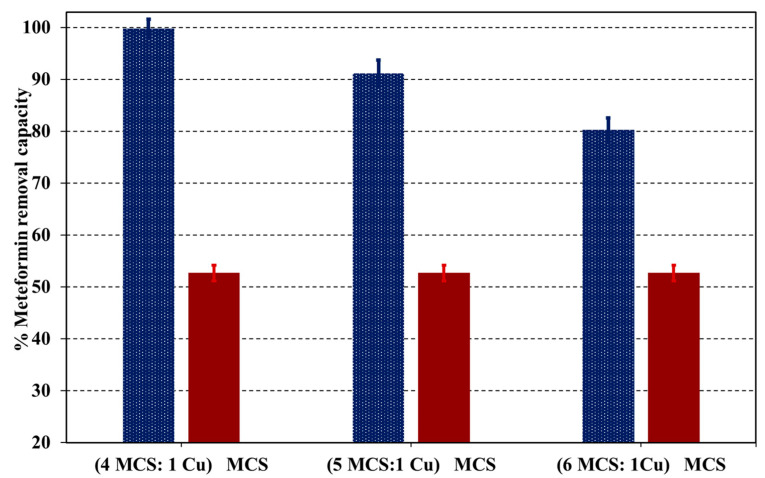
The impact of immobilized Cu-NPs on the removal capacity for the metformin–Cu@MCS adsorption system.

To assess the performance of adsorption of Cu-NPs in the magnetic chitosan nanocomposite, sorption tests were conducted for both MCS and Cu@MCS nanocomposites at pH = 6.0. Figure 6 displays the findings of this comparison investigation. The capacity to remove metformin was considerably increased by 43.5% by the supported Cu nanoparticles.

Two key variables could account for this significant increase in adsorption, which is mostly attributed to Cu-NPs. The first reason is that the MCS moiety is prevented from being aggregated, which significantly affects the Cu@MCS’s surface area. The formation of Cu–metformin complexes onto the surface of the Cu@MCS nanocomposite in the pH range of 4–8 is the second aspect. These findings show that the developed Cu@MCS nanocomposite’s capacity to eliminate metformin is significantly influenced by Cu-NPs.

### 3.5. Sorption Mechanisms

**Figure 7:**
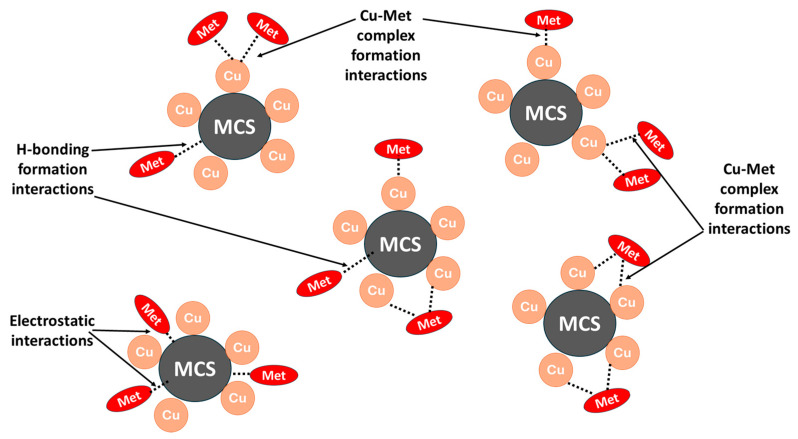
A schematic diagram depicting the proposed interaction mechanisms between metformin and Cu@MCS nanocomposite.

The sorption of metformin and Cu@MCS can be described using four distinct types of interactions, depending on the pH of the solution [74,75]. In the first, Cu-NPs bind with the two free imine (-C=NH) groups to form stable five-membered chelate rings, as well as bind with the free amino group to form Cu–metformin surface complexes. In addition, the Cu-NPs prevent the MCS moiety from being aggregated, which significantly affects the surface area of the Cu@MCS and, in turn, enhances the metformin removal capacity. The investigations in Section 3.3 prove that the primary interactions between the Cu-MCS and the metformin are the surface Cu-complex formation [76]. The second method is the hydrophobic interaction between the two methyl groups on the 3°-N-atom of metformin with the magnetite in MCS. The third one has to do with the H-bonding that forms between the MCS’s several oxygen-containing groups (hydroxyl groups of CS and negatively charged oxygen atoms of magnetite) and the N-functional groups in metformin. The fourth method involves electrostatic interactions, which are variable with the solution pH. These interactions are mostly brought on by the attraction between the positively charged protonated nitrogen groups of metformin and the negatively charged groups in the MCS [75,77,78]. Certainly, the immobilization of Cu-NPs in the Cu@MCS nanocomposite has a crucial advantage for improving metformin’s adsorptive removal. Figure 7 presents a schematic illustration of the proposed interactions between metformin and the Cu@MCS nanocomposite.

### 3.6. Sorption Kinetics

**Figure 8:**
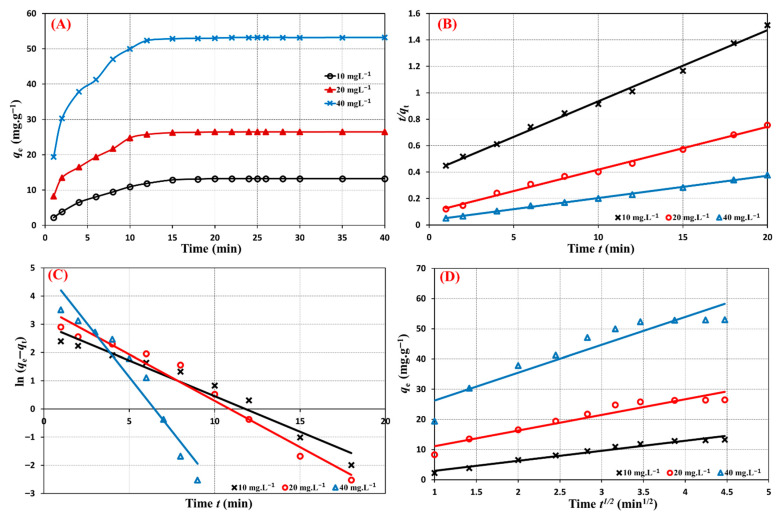
Contact time effect (**A**), PSO (**B**), PFO (**C**), and intraparticle (**D**) kinetic models for metformin adsorption removal by Cu@MCS nanocomposite for different initial concentrations (10, 20, 40 mg·L^−1^) at 298 K.

The influence of contact time on the metformin adsorptive uptake by the Cu@MCS was examined in this study using three distinct initial concentrations (10, 20, and 40 mg∙L^−1^). The three concentrations’ metformin adsorption capabilities are shown against contact time in Figure 8A. The equilibrium between the binary nanocomposite and metformin is reached after 20 min, and the adsorption process proceeds in two consecutive phases. In just five minutes, the first kinetics adsorption stage is completed, while the second step takes ten minutes to complete. For the three distinct starting concentrations, the same pattern is seen. The linear plots of pseudo-second order (PSO), pseudo-first order (PFO), and intraparticle models were used to assess the fitness of the experimental data to assess the kinetics data of the Cu@MCS–metformin adsorption system (Figure 8B–D). Table 1 displays the linear kinetic sorption equations and fitting parameters for the applied models. It shows higher correlation coefficients (*R*^2^ = 0.995−0.998) of the PSO rate expression for all examined initial doses of metformin (Figure 8B). The experimental findings (18.20, 31.42, and 60.15 mg g^−3^) closely align with the adsorption capacity values derived from the PSO model. This confirms that the Cu@MCS nanocomposite removes metformin from water in a rate-controlled manner through chemisorption, just like it does for several other pharmaceutical pollutants [79,80].

### 3.7. Reusability of Cu@MCS Nanocomposite

**Figure 9:**
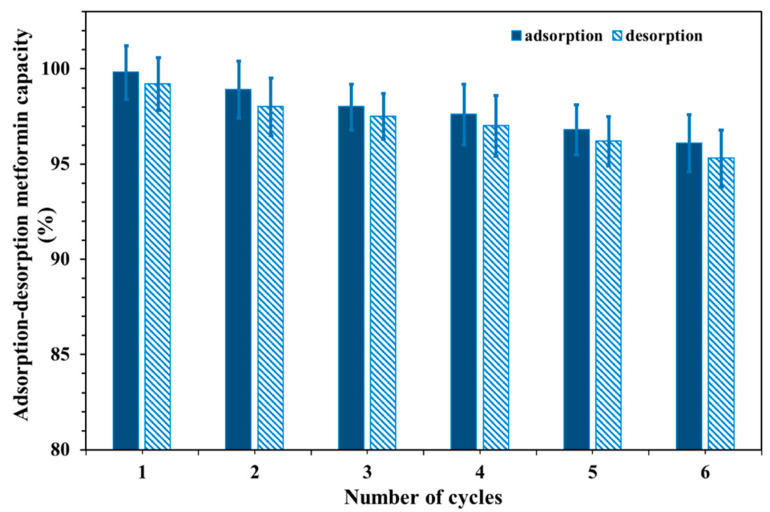
Recyclability of the metformin–Cu@MCS adsorption system.

For adsorbents to be useful, they need to be reusable and have a suitable amount of capacity to adsorb. Figure 8 displays the results of this work, which demonstrates that a 0.15 *M* HCl solution could desorb almost all the metformin attached to the surface of the Cu@MCS. The desorbed metformin remained consistent for a maximum of six adsorption–desorption cycles. After six cycles, the adsorption capacity started to decrease. Once the recycled Cu@MCS nanocomposite was extracted, its weight was measured. After regenerating the Cu@MCS nanocomposite six times, we found no evidence of substantial mass loss. Moreover, the range of metformin extraction percentages from the recycled Cu@MCS was determined to be comparable to that of the original Cu@MCS nanocomposite. Results of metformin removal efficiencies ranging from 99.8% to 96.1% in the sixth and first cycles of desorption showed that the Cu@MCS nanocomposite’s sorption ability did not substantially decline after six runs (Figure 9). Moreover, FTIR analysis after six regeneration cycles (Figure S2) showed that the characteristic functional groups of Cu@MCS remained intact, confirming the preservation of structural integrity. In addition, no significant peak shifts or intensity losses were observed, indicating that neither repeated HCl regeneration nor pH/ionic strength variations caused detectable chemical degradation of the chitosan backbone, Cu–O, or Fe–O bonds. The remarkable desorption efficiency and reusability of Cu@MCS suggest that the nanocomposite could be a useful, cost-effective nanosorbent for the extraction of metformin from water.

### 3.8. Sorption Isotherms

**Figure 10:**
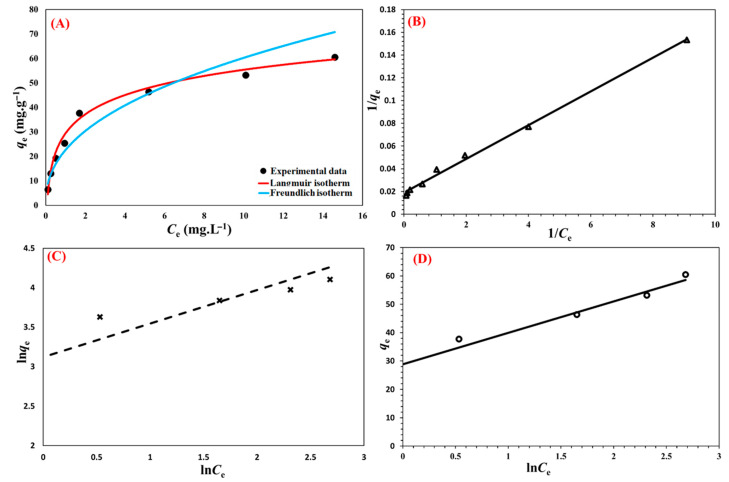
Nonlinear fit of isotherm models (**A**) and linear plots of Langmuir (**B**), Freundlich (**C**), and Temkin adsorption (**D**) isotherm models for the uptake of metformin by Cu@MCS nanocomposite at 298.15 K.

Understanding of the adsorbent’s affinity, surface properties, and adsorption mechanisms may be gained from the isotherm of metformin sorption onto the Cu@MCS nanosorbent. The adsorption process was examined using the Freundlich, Langmuir, and Temkin adsorption models (Figure 10). These models’ linear shapes are depicted as follows:

(3) $$\frac{1}{q_{e}}\;=\;\frac{1}{q_{m}}\;+\;\frac{1}{K_{L}\;q_{m}\;C_{e}}$$

(4) $$\ln\;q_{e}\;=\;\ln K_{F}\;+\;\frac{1}{n}\;\ln\;C_{e}$$

(5) $$\;q_{e}\;=\;K_{T}\ln C_{e}\;+\;K_{T}\;\ln\;f$$

where *C*_e_ is the metformin’s equilibrium concentration (mg L^−1^); *q*_m_ (mg g^−1^) is the nanocomposite’s maximum adsorption capacity; *n* represents the parameter of the Freundlich linearity, which establishes if the sorption procedure is preferred; and *K*_L_, *K*_F_, and *K*_T_ are the three models’ sorption constants. The Langmuir model provides the best description of a reversible sorption process with the formation of a monolayer on a homogeneous adsorbent, while the formation of multilayers is suggested by the empirical Freundlich model. According to the Temkin model, the chemisorption process is characterized by powerful electrostatic forces between negative and positive charges [80]. Table 2 lists these models’ adsorption isotherms, along with the fitting parameters for each. The Temkin model (0.9881) and the Freundlich model (0.9395) largely fit the experimental data, whereas the correlation coefficients (*R*^2^) of metformin for Langmuir (0.9963) fit the data quite well. As a model fitting parameter, the theoretical maximum adsorption capacity (*q*_m_) for metformin was determined to be 52.91 mg g^−1^. The proportion of metformin’s deprotonated amine and imine structural groupings is greater at the experimental pH of 6.0, which promotes complexation with the immobilized Cu-NPs and improves surface adsorption as well as the hydrogen bonding, electrostatic, and π-π EDA interactions. The Cu@MCS nanocomposite’s maximum adsorption capacities for metformin are compared to those of other adsorbents in Table 2. Cu-NPs functionalize the MCS in the developed nanocomposite, giving it a distinct advantage. By forming surface inner complexes with the supporting copper nanoparticles, these innovative moieties significantly increase the *q*_m_ values. Table 3 displays the results of the literature review on the maximum adsorption capacities of several adsorbents for metformin. The literature review indicates that the expected *q*_max_ value for metformin adsorption in this work is similar to the values reported in the literature for metformin adsorption on various adsorbents; nevertheless, Cu@MCS is distinguished by rapid metformin adsorption at ambient pH and temperature. The results of this investigation suggest that the Cu@MCS nanocomposite could be an excellent candidate for use in real systems.

### 3.9. Analytical Performance of Cu@MCS Nanocomposite

The metformin adsorption capacity of the Cu@MCS was evaluated by conducting sorption tests for the removal of metformin in samples of tap water, wastewater, river water, and bottled drinking water. The study’s findings are listed in Table 4. Compared to samples of spiked bottled drinking and tap water, the percentages of metformin removal efficiency from wastewater and river water (99.5–99.9%) were significantly greater. These promoting effects are most likely related to the humic materials in river water and pH differences [87,88].

In natural aquatic systems, the presence of chloride, carbonate, sulfate, sodium, calcium, magnesium ions, and dissolved oxygen can significantly influence the stability of Cu-NPs. It is important to note, however, that the Cu@MCS adsorbent is not composed of free metallic copper alone but rather Cu-NPs immobilized within the CS framework via coordination with hydroxyl (-OH) and amino (-NH_2_) functional groups. This immobilization minimizes direct interaction between copper species and the bulk solution, thereby enhancing stability, preserving surface-active states, and preventing uncontrolled leaching or corrosion. Consistent with this stabilization mechanism, our experiments demonstrated negligible copper release (<0.5 mg∙L^−1^) even after multiple adsorption–desorption cycles, confirming the structural and functional robustness of the material.

Regarding complex environmental matrices such as wastewater and river water, these typically contain suspended solids, microorganisms, and diverse organic and inorganic constituents. To ensure comparability, all samples in this study were filtered through 0.45 µm membranes prior to testing, thereby eliminating coarse particulates. Although microbial and colloidal fouling are recognized challenges in long-term applications, the present work was designed as a controlled batch investigation to demonstrate proof-of-concept adsorption under environmentally relevant chemical conditions, rather than to evaluate large-scale deployment. The consistently high recovery observed can thus be attributed to experimental design and controlled background conditions.

In terms of selectivity, despite the heterogeneous organic composition of wastewater and river water, the remarkable efficiency of metformin removal is attributable to two primary factors: (i) the strong coordination affinity between metformin’s amine groups and copper binding centers and (ii) the cooperative role of natural organic matter in facilitating co-adsorption and π–π interactions. This mechanistic interpretation is further supported by comparative analyses across different water matrices and corroborated by the pH and conductivity data presented in Table 4.

## 4. Conclusions

A copper-functionalized magnetic chitosan nanocomposite (Cu@MCS) was developed and utilized for the first time to achieve rapid and highly efficient elimination of metformin from environmental water. The embedded Cu-NPs enhanced the material’s performance in two major ways: (i) they improved the dispersion of the composite and preserved its two-dimensional architecture by preventing the aggregation of magnetite particles, and (ii) they promoted strong binding of metformin through the formation of Cu–metformin surface complexes. The adsorption pathway was found to be pH-sensitive and governed by four dominant interactions: hydrogen bonding between metformin amine groups and oxygenated functionalities of the nanocomposite, π–π donor–acceptor interactions with the aromatic domains of chitosan, cation–π interactions, and, most importantly, surface complexation with Cu-NPs. Kinetic evaluation fitted well to the pseudo-second-order model, highlighting the rapid nature of the adsorption process. Under optimized conditions, Cu@MCS removed metformin with a remarkable efficiency of 99.8 ± 2.7% across diverse water matrices. These findings demonstrate that f combines superior adsorption capacity, rapid kinetics, high reproducibility, strong enrichment ability, and excellent reusability, making it a promising adsorbent for pharmaceutical contaminant removal.

## Figures and Tables

**Table 1:** The linear kinetic equations and parameters for the adsorption of metformin onto Cu@MCS nanocomposite at pH 6 for 15 mg mass dosage.

Kinetic Model/Linear Equation	Kinetic Parameters	10.0 mg∙L^−1^	20.0 mg∙L^−1^	40.0 mg∙L^−1^
	*q*_e, exp._ (mg g^−1^)	18.20	31.42	60.15
*Pseudo-second order*				
$\frac{t}{q_{t}}=\;\frac{1}{k_{2}q_{e}^{2}}+\frac{t}{q_{e}}$	*q*_e, calc._ (mg g^−1^)	18.59	30.86	59.17
	*K*_2_ (g mg ^−1^ min ^−1^)	7.0 × 10^−3^	0.011	0.008
	*R* ^2^	0.9952	0.9953	0.9980
*Pseudo-first order*				
$\ln\;(q_{e}-q_{t})=\ln\;q_{e}-k_{1}t$	*q*_e, calc._ (mg g^−1^)	19.63	35.74	143.5
	*K*_1_ (min^−1^)	0.252	0.329	0.768
	*R* ^2^	0.9541	0.9691	0.9349
*Intraparticle diffusion*				
$q_{t}=K_{p}\;t^{0.5}+c$	*K*_p_ (mg·g^−1^·min^−1/2^)	7.345	3.307	9.206
	*C*	3.643	0.343	17.09
	*R* ^2^	0.9909	0.9688	0.8868

**Table 2:** Parameter fitting of linear plots for Temkin, Freundlich, and Langmuir sorption isotherms for the Cu@MCS nanocomposite’s sorptive removal of metformin.

Langmuir			Freundlich			Temkin		
*q*_m_ (mg g^−1^)	*K*_L_ (L mg^−1^)	*R* ^2^	*K*_F_ (L mg^−1^)	*n*	*R* ^2^	*K*_r_	*f*	*R* ^2^
52.91	1.268	0.9963	3.0 × 10^12^	0.0900	0.9395	11.10	13.40	0.9881

**Table 3:** Comparison of the Cu@MCS nanocomposite with other nanosorbents for metformin removal in the literature.

Adsorbent	Adsorption Parameters	*q*_max_	Reference
Multi-walled carbon nanotubes, commercial	pH = 8.20, T = 25 °C	79.94	[81]
Graphene oxide	pH = 6; contact time 160 min T = 15, 30, and 45 °C.	96.748, 89.099, 88.517	[82]
Fe-ZSM-5 nano-adsorbent	Contact time 20 min. T = 25 °C	14.992	[83]
Activated carbon from orange peel	pH = 7, contact time 240 min, T = 50 °C	50.99	[84]
Activated carbon from agricultural waste	pH = 7, contact time 125 min, T = 20 °C	44.84	[85]
Water-treated clay	pH = 6, contact time 30 min, T = 25 °C	25.268	[86]
Cu@MCS	pH = 6, contact time 20 min, T = 25 °C	52.91	The present study

**Table 4:** Metformin’s adsorptive removal from environmental water samples using the Cu@CGO nanocomposite.

Water Sample	Sample Parameters		Conc. Added (mg L^−1^)	Conc. Found (mg L^−1^)	Recovery (%)
	pH	Conductivity (μs cm^−1^)			
River water	7.83	324.0	5.0	4.98	99.7 ± 4.2
			10.6	10.59	99.9 ± 3.7
			20.0	19.96	99.8 ± 4.5
Wastewater	9.05	304.7	5.3	5.29	99.8 ± 4.1
			10.2	10.18	99.8 ± 3.5
			20.0	19.92	99.6 ± 3.7
Tap water	7.13	81.3	5.0	4.98	99.7 ± 2.7
			10.4	10.36	99.6 ± 3.0
			20.3	20.20	99.5 ± 2.5
Bottled water	8.62	24.15	10.2	10.17	99.7 ± 2.7

## Data Availability

The original contributions presented in this study are included in the article/Supplementary Material. Further inquiries can be directed to the corresponding author.

## Supplementary Materials

The following supporting information can be downloaded at https://www.mdpi.com/article/10.3390/polym17223046/s1, Figure S1: The nitrogen adsorption-desorption hysteresis loop of the Cu@MCS nanocomposite at 77.04 K; Figure S2: FTIR of the Cu@MCS nanocomposite before and after six adsorption-desorption cycles.
