# Supplementary material for: Synergistic Impact of Copper Nanoparticles Functionalized with Magnetic Chitosan on the Enhanced Adsorptive Sequestration of Metformin Diabetic Drug from Environmental Samples
Source: Polymers (Basel). 2025 Nov 17;17(22):3046. doi: 10.3390/polym17223046 (PMC12656049; doi:10.3390/polym17223046)

<u>Supplementary Materials</u>:

Figure S1: The nitrogen adsorption-desorption hysteresis loop of the Cu@MCS nanocomposite at 77.04 K

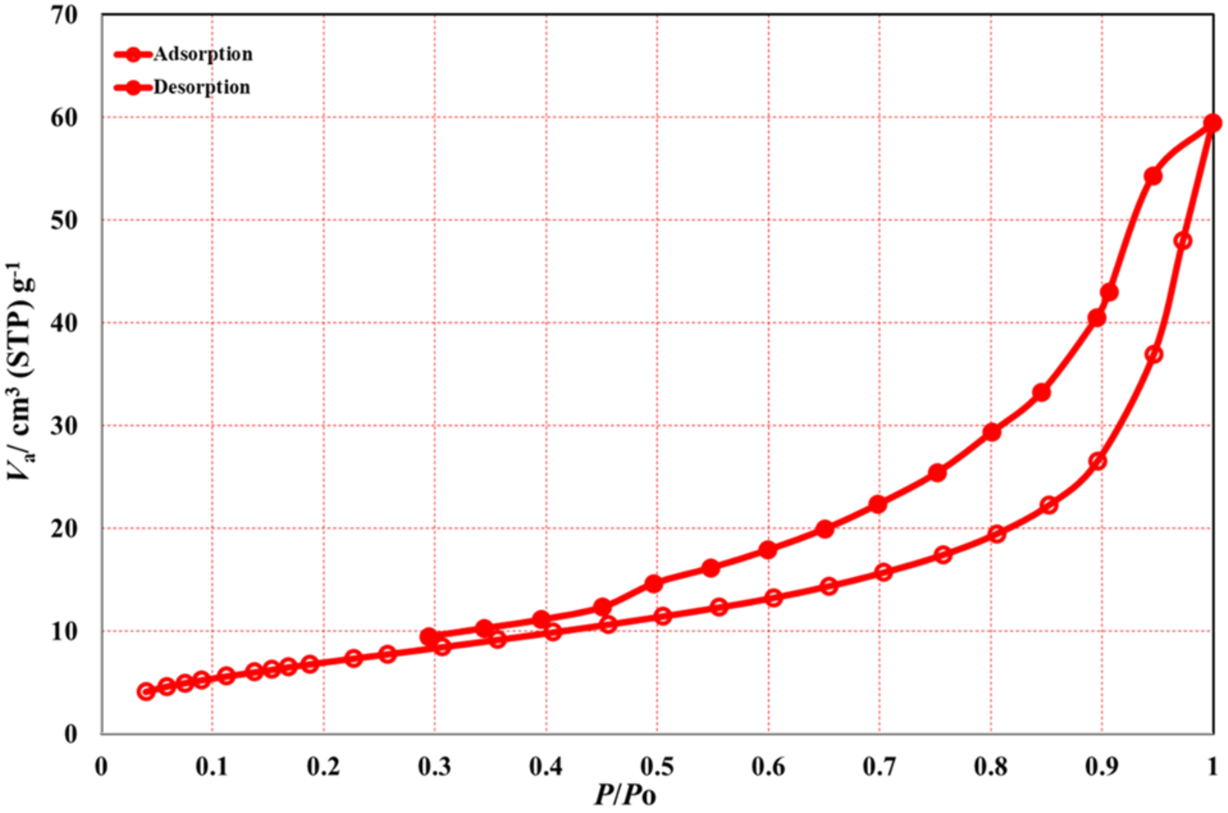

Figure S2: FTIR of the Cu@MCS nanocomposite before and after six adsorption-desorption cycles

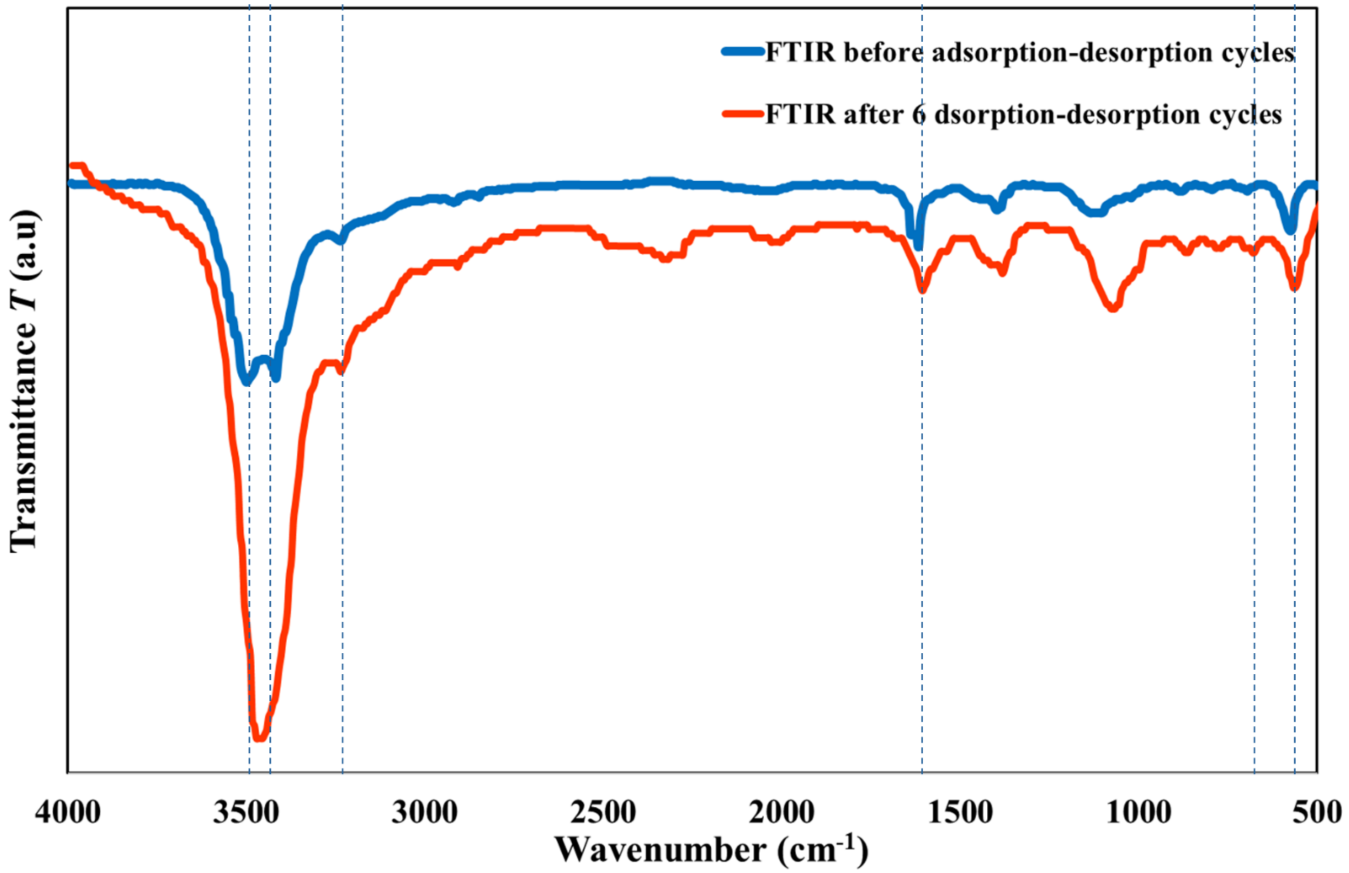

Supplement: Supplementary file 1 [file polymers-17-03046-s001.zip › polymers-3895850-supplementary.pdf]
